# Quantum Mechanics and Its Evolving Formulations

**DOI:** 10.3390/e23010124

**Published:** 2021-01-19

**Authors:** Jean-Pierre Antoine

**Affiliations:** Institut de Recherche en Mathématique et Physique, Université catholique de Louvain, B-1348 Louvain-la-Neuve, Belgium; jean-pierre.antoine@uclouvain.be

**Keywords:** quantum mechanics, von Neumann, bra-ket formalism, rigged Hilbert space

## Abstract

In this paper, we discuss the time evolution of the quantum mechanics formalism. Starting from the heroic beginnings of Heisenberg and Schrödinger, we cover successively the rigorous Hilbert space formulation of von Neumann, the practical bra-ket formalism of Dirac, and the more recent rigged Hilbert space approach.

## 1. Introduction: The Pioneers

By the year 1900, physics was considered to be finished by the main experts, such as Lord Kelvin or Lord Rayleigh. Newton’s classical mechanics and Maxwell’s electromagnetism reigned over the whole branch; only details remained to be improved. In addition, large systems were described by thermodynamics, developed by Maxwell and Boltzmann on a statistical basis. Only some rare phenomena resisted an explanation. However, a revolution was about to explode.

One case that could not be predicted correctly was the black-body spectrum—that is, the spectrum of electromagnetic radiation emitted by a heated body. Then, in 1900, Planck explained it by introducing artificial entities, called *quanta*, that were supposed to be elementary constituents of energy, each of them proportional to a given frequency. This made energy discrete. Then, in 1905, Einstein explained the photoelectric effect as the collision between an atom and a particle. Thus, light is made of elementary grains, called photons, in fact Planck’s quanta; light is also corpuscular, not only a wave phenomenon (he got a Nobel Prize in 1921 for this achievement). In the same year 1905 (called “annus mirabilis”), he overturned classical Galilean mechanics with his special relativity, making it compatible with electromagnetism.

Then, in 1913, Bohr introduced his planetary model of the atom, building on the experiments of Rutherford. This, however, generated a paradox. The model says that an electron orbiting the nucleus of the atom can have only a discrete set of binding energies; the latter are quantized. In addition, the electron may jump from one “orbit” to a lower one, with the energy difference being emitted as a photon. While this fact fits with experimental data, it is incompatible with classical mechanics: a classical electron would spiral around the nucleus, loosing energy in a continuous matter. Slightly later, Sommerfeld expanded Bohr’s model by allowing elliptical orbits (Bohr had only circular ones), but this did not resolve the paradox.

A first hint to a solution was provided by Heisenberg in 1925 [1], who, together with Born and Jordan, introduced matrix mechanics. Actually, Heisenberg was manipulating arrays of numbers, without knowing that mathematicians had formalized this before. As Born recalled,

One morning about the 10 July 1925 I suddenly saw light: Heisenberg’s symbolic manipulation was nothing but the matrix calculus well-known to me since my student days [2].

This approach opened the way to an algebraic formulation of quantum mechanics, that flourished much later in statistical mechanics.

A second attack on the problem is wave mechanics, introduced by Schrödinger in 1926 [3]. The idea was to introduce a wave equation, inspired by the classical analysis of waves, that takes the form of an eigenvalue problem for the Hamiltonian. The latter is obtained from its classical expression by certain correspondence rules. The task of the physicist is to solve the equation—that is, to find the eigenvalues, which are the allowed values of the energy.

In a third paper, Schrödinger showed, albeit in a somewhat heuristic way, that the two approaches are equivalent [4]. Dirac, in turn, proved in 1930 that the equivalence of the two theories results from his transformation theory [5].

In parallel to these two supposedly equivalent formulations of the theory, another set of controversies emerged concerning its interpretation. Based on a statistical approach, largely due to Born, and the notion of noncommuting “observables”, which leads to Heisenberg’s uncertainty relations, the so-called Copenhagen interpretation gradually took over, although there are always oppositions (Einstein, for one, never admitted it: “God does not play dice!”). In fact, most physicists use it, more or less explicitly, but there are still exceptions, even today.

Clearly, the situation was rather messy. As Gottfried puts it [6]: “The old quantum theory was a diabolically clever hodge-podge of classical laws and seemingly unrelated ad hoc recipes.” The mathematics is not well defined, one uses eigenfunctions of infinite norm, as well as the so-called delta “function”, operators are treated in a formal way, and so on. Clearly, the time was ripe for a precise mathematical overhaul of the whole theory, and this was the achievement of John von Neumann [7].

## 2. Von Neumann’s Axiomatics

The first thing to do was obviously to define rigorously the mathematical framework. To that effect, von Neumann begins his book with a long chapter (170 pages) on the abstract Hilbert space. This is in fact the first complete definition of a Hilbert space (Hilbert himself had only considered a special case, namely the space $\ell^{2}$ of square integrable sequences). All the basic ingredients are there: a vector space equipped with an inner product and the corresponding norm, plus the requirement of completion. Then operators, bounded or unbounded, unitary operators, self-adjoint operators (which he calls “hypermaximal”), trace class (also called nuclear) and Hilbert–Schmidt operators, orthogonal projections, and the spectral theorem for self-adjoint operators. This pioneering work may well be von Neumann’s essential contribution.

Next, von Neumann turns to quantum mechanics (QM). However, he never gives a precise list of its axioms. Instead, he bases everything on the statistical affirmations of QM; thus, he endorses the probabilistic interpretation of Born–Jordan–Dirac. The starting point is the following postulate.

Take a system in the state $\phi =\phi \left(q_{1},q_{2},\ldots ,q_{k}\right)\in L^{2}\left(R^{3k}\right)$. Then, the probability that the system is in the volume *V* of configuration space is given by

$$\;\int \int \;\ldots \int_{V}{\left|\phi \left(q_{1},q_{2},\ldots ,q_{k}\right)\right|}^{2}\;\;dV.$$

In other words, $|\phi \left(q_{1},q_{2},\ldots ,q_{k}\right){|}^{2}$ is the probability density.

From this statement, von Neumann derives the whole structure of QM: expectation values, statistical operator, measurability and simultaneous measurability, uncertainty relations, and so on.

At this stage, one may formalize the rules (axioms) of QM as follows.

1. The space of states of a system is a Hilbert space H.
  In particular, the requirement that H be complete offers a certain mathematical “comfort”—that is, powerful theorems that do not hold without it.
2. A state is represented by a ray ϕ∈H (“pure state”: ${∥\phi ∥}_{}=1$) or a density operator ρ, i.e., a trace class operator ρ>0 with Trρ = 1; in particular, ρ defines a pure state if and only if one has $\rho^{2}=\rho$, i.e., ρ is a projection.
3. A physical observable is represented by a self-adjoint operator in H. Therefore, one may use
  . The spectral theorem for self-adjoint operators.
  . Stone’s theorem: $e^{iAt}$ is unitary if and only if $A=A^{*}$; this is the key of the time evolution.
4. Given a physical observable represented by the self-adjoint operator *A*, its expectation value in the state ρ is given by
  $${\langle A\rangle }_{\rho }=\mathrm{Tr}\;\rho \;A.$$
  For a pure state ϕ, this becomes
  $${\langle A\rangle }_{\phi }=\langle \phi |A\;\phi \rangle .$$
5. Given two states ϕ,ψ∈H, the transition probability ϕ→ψ is given by
  $$P\left(\phi \to \psi \right)={\left|\langle \psi |\phi \rangle \right|}^{2}.$$

Before continuing, we would like to point out a recent book [8] that provides a thorough analysis of the space of density matrices (finite dimensional Hilbert–Schmidt operators) and its geometry. This space is in fact a Hilbert space with the trace inner product $\langle A|B\rangle =\mathrm{Tr}\;A^{*}B$.

This formulation is rigorous, but several problems remain, namely,

- Not all vectors in H may represent states.
  First, some states ϕ∈H cannot be prepared experimentally, e.g., a state of “infinite energy”, i.e., ϕ∉D(H), if the Hamiltonian *H* is unbounded. Worse, some vectors cannot make sense as states. For instance, there might be superselection rules; what meaning can be given to $\phi_{\mathrm{boson}}+\psi_{\mathrm{fermion}}$? More basically, there is no plane wave in H.
- Which self-adjoint operators in H can possibly represent an observable?
  Certainly not all of them (Pauli was the first to note this). For instance, what could represent the operator A+B if *A* and *B* do not commute? Wigner kept asking: what does the operator $A=p^{2}+q^{2}$ represent for a hydrogen atom?
  More generally, the question is how to select the “good” operators. This asks for a precise definition of the concept of observable, containing two aspects: the physical definition—that is, how the quantity can be measured experimentally—and the mathematical definition of the representative operator.
- There are problems with unbounded operators: one has to make their domain precise; they cannot be added freely. Similarly, operators with a continuous spectrum have the problem that there are no eigenvectors corresponding to points of the continuous spectrum. Another difficulty is that the transition between the Schrödinger picture and the Heisenberg picture may be problematic if the Hamiltonian is unbounded.

## 3. Dirac’s Axiomatics

However, such problems do not perturb the vast majority of physicists, who simply ignore von Neumann and prefer to use the simpler language of Dirac [5]. The same situation prevails for most textbooks. Here is a list of some popular ones, arranged in chronological order: Messiah [9], Merzbacher [10], Bohm [11], Sakurai [12], Ballentine [13], Gottfried [6], Le Bellac [14], Cohen-Tannoudji et al. [15].

Of course, there are exceptions; some textbooks are more mathematically flavored—for instance, those of Prugovečki [16] or Isham [17]. An extreme opposite attitude is that of Pauli, who once wrote in a letter to Born (19 July 1925) “You are only going to spoil Heisenberg’s physical ideas with your futile mathematics!”

### 3.1. The “Bra-Ket” Formalism

The formalism may be synthesized in four “axioms”:

1. The states of a quantum system constitute a vector space E, equipped with a Hermitian inner product 〈·|·〉, which manifests the superposition principle, and there is a bijective correspondence
  bra〈ϕ|↔ket|ϕ〉.
2. Given two states ϕ,ψ∈E, the transition probability ϕ→ψ is given by
  $$P\left(\phi \to \psi \right)={\left|\langle \psi |\phi \rangle \right|}^{2}.$$
3. Each physical observable is represented by a linear Hermitian operator on E, and these operators form a (non-Abelian) algebra: A+B and AB are well defined.
4. Each physical system has a complete system of commuting observables (CSCO), and the eigenvectors of the elements of this CSCO constitute a basis of E. Thus, every state ψ∈E may be expanded into that basis.

### 3.2. Spectral Analysis

Let $\left\{\lambda_{i},\lambda \right\}$ be the spectrum of the CSCO and $\left\{|\xi_{\lambda }\equiv |\lambda_{i}\rangle ,|\lambda \rangle \right\}$ the common eigenvectors. Then one has:

- Orthogonality relations
  $$\begin{array}{cc} \; & \langle \lambda_{i^{\prime }}|\lambda_{i}\rangle =\delta_{i^{\prime }i}\;\left(\mathrm{discrete}\;\mathrm{spectrum}:\;\mathrm{normalizable}\;\mathrm{eigenvectors}\right).\\ \; & \langle \lambda^{\prime }|\lambda \rangle =\delta \left(\lambda^{\prime }-\lambda \right)\;\left(\mathrm{continuous}\;\mathrm{spectrum}:\;\mathrm{nonnormalizable}\;\mathrm{eigenvectors}\right). \end{array}$$
- Closure relation:
  $$I=\int_{R}\;|\xi_{\lambda }\rangle \langle \xi_{\lambda }|\;\;d\mu \left(\lambda \right)=\sum_{i}|\lambda_{i}\rangle \langle \lambda_{i}|+\int_{R}\;|\lambda \rangle \langle \lambda |\;\;d\lambda ,$$
  that is, $\;d\mu \left(\lambda \right):=\sum_{i}\delta_{\lambda_{i}}+\;d\lambda$, where dλ is a suitable measure on the Hilbertian continuous spectrum. Thus, $|\xi_{\lambda }\rangle \langle \xi_{\lambda }|$ may be interpreted as the “projection” on the state $|\xi_{\lambda }\rangle$.
- Expansion of an arbitrary state ϕ∈E:
  $$|\phi \rangle =\sum_{i}|\lambda_{i}\rangle \langle \lambda_{i}|\phi \rangle +\int_{R}|\lambda \rangle \;\langle \lambda |\phi \rangle \;\;d\lambda .$$
- Example: take the CSCO *H* (rotation invariant Hamiltonian), $J^{2}$, $J_{3}$ (angular momentum), with common eigenvectors $|Ejj_{3}\rangle$:
  $$H|Ejj_{3}\rangle =E|Ejj_{3}\rangle ,\;J^{2}|Ejj_{3}\rangle =j\left(j+1\right)|Ejj_{3}\rangle ,\;J_{3}|Ejj_{3}\rangle =j_{3}|Ejj_{3}\rangle .$$
- Probabilistic interpretation, like von Neumann’s:
  $${\langle A\rangle }_{\phi }=\langle \phi |A|\phi \rangle \;\mathrm{if}\;\langle \phi |\phi \rangle =1,\;\mathrm{and}\;\mathrm{so}\;\mathrm{on}.$$

### 3.3. What about a Rigorous Dirac Formalism?

All this is practical and used by most physicists, but it is not correct in a Hilbert space. Therefore, E cannot be taken as a Hilbert space H… What is it then? How can one reconcile the two approaches? A solution is to work in a Gel’fand triplet (rigged Hilbert space). This approach was proposed independently by Roberts [18,19], the author [20,21,22] and Bohm [23]. We will discuss it in the next section.

## 4. The RHS Approach

### 4.1. Building a Rigged Hilbert Space

- Stage #1: Select a family O of labeled observables that in fact define the system, i.e., (i) observables with a precise physical interpretation, e.g. in terms of measurement (position, energy, …) and (ii) represented by self-adjoint operators that possess a common invariant dense domain D⊂H: AD⊂D,∀A∈O. Thus, O is an algebra.
- Stage #2: Equip D with a suitable topology that makes all operators A∈O continuous. In this way, one obtains a topological vector space Φ dense in H, with continuous embedding ı:Φ→H (such a topology may be defined by the family O itself).
- Stage #3: By (anti)duality, one obtains the triplet (RHS):
  (1) $$\Phi \subset H\subset \Phi^{\times }$$
  where $\Phi^{\times }$ is the conjugate dual of Φ, i.e., the space of continuous conjugate linear functionals on Φ (this ensures that both natural embeddings Φ→H and $H\to \Phi^{\times }$ are linear).

### 4.2. More about the RHS

A natural interpretation of the elements of the triplet (1) runs as follows:

. Φ represents those states that are physically preparable.
. H is von Neumann’s Hilbert space.
. $\Phi^{\times }$ contains generalized states associated with measurement operations.

As for mathematical properties, one requires that Φ be

. complete, i.e., every Cauchy sequence converges to an element of Φ; otherwise, its completion might fail to be contained in H.
. reflexive: $\Phi^{\times \times }=\Phi$, so that no other space than Φ and $\Phi^{\times }$ has to be considered, which would ruin the interpretation above.
. nuclear, which allows to apply the generalized spectral theorem of Gel’fand–Maurin.

This demands a definition. In the simplest case, assume Φ is the intersection of a decreasing scale of Hilbert spaces

$$\Phi =\cap_{n\in N}H_{n},\ldots \subset \;H_{3}\;\subset \;H_{2}\;\subset \;H_{1}\;\subset \;H.$$

In technical terms, Φ is a projective limit $\Phi =\;\lim^{⟵}H_{n}\;,$ with the corresponding locally convex topology.

Then Φ is said to be nuclear if, for each n∈N, there is a m∈N,m>n, such that the natural embedding $H_{m}\to H_{n}$ is a Hilbert–Schmidt operator. The classical example is the Schwartz space S(R) of smooth fast decreasing functions.

## 5. The Generalized Spectral Theorem of Gel’fand–Maurin

### 5.1. Eigenvectors

Let *A* be a closed operator in H, continuous from Φ to Φ. Then, one defines the adjoint

$A^{\times }:\Phi^{\times }\to \Phi^{\times }$, extension of the Hilbertian adjoint $A^{†}$, by

$$A^{\times }F\left(\phi \right)=F\left(A\phi \right),\;\mathrm{for}\;\mathrm{all}\;\phi \in \Phi \;\mathrm{and}\;F\in \Phi^{\times },$$

or, equivalently,

$$\langle \phi |A^{\times }F\rangle =\langle A\phi |F\rangle ,\;\mathrm{for}\;\mathrm{all}\;\phi \in \Phi \;\mathrm{and}\;F\in \Phi^{\times }.$$

A vector $\xi_{\lambda }\in \Phi^{\times }$ is called a generalized eigenvector of *A*, with eigenvalue λ∈C, if one has

$$\langle \phi |A^{\times }\xi_{\lambda }\rangle \equiv A^{\times }\xi_{\lambda }\left(\phi \right)=\lambda^{*}\xi_{\lambda }\left(\phi \right)\equiv \lambda^{*}\langle \phi \xi_{\lambda }\rangle ,\;\forall \;\phi \in \Phi .$$

Using Dirac’s notations, this becomes

$$A^{\times }|\xi_{\lambda }\rangle =\lambda^{*}|\xi_{\lambda }\rangle ,\;|\xi_{\lambda }\rangle \in \Phi^{\times }.$$

Actually, for obtaining a complete parallel with Dirac’s bra-ket formalism, one needs to consider a second RHS, namely:

(2) $$\Phi \subset H\subset \Phi^{\prime },$$

where $\Phi^{\prime }$ denotes the dual of Φ, i.e., the space of continuous linear functionals on Φ. Then, whereas $\Phi^{\times }$ contains the ket vectors, $\Phi^{\prime }$ contains the bra vectors, and indeed the two are in one-to-one correspondence. For a pedagogical description of this formulation, one may consult [24].

### 5.2. The Generalized Spectral Theorem

Assume

. *A* has a self-adjoint extension $A_{0}$ in H; thus, $A^{\times }$ is an extension of *A* et $A_{0}$ (collectively, $\hat{A}$)
. Φ is nuclear and complete.

Then, *A* (or $\hat{A}$) possesses a complete orthonormal system of generalized eigenvectors $\xi_{\lambda }\in \Phi^{\times },\;\lambda \in R$, i.e., one has, for all ϕ,ψ∈Φ and a suitable measure μ:

$$\begin{array}{cc} \langle \phi |\psi \rangle & =\int_{R}\;\xi_{\lambda }\left(\phi \right)\;{\xi_{\lambda }\left(\psi \right)}^{*}\;\;d\mu \left(\lambda \right)\\ \; & \equiv \int_{R}\;\langle \phi |\xi_{\lambda }\rangle \;\langle \xi_{\lambda }|\psi \rangle \;\;d\mu \left(\lambda \right)\\ \; & =\sum_{i}\langle \phi |\lambda_{i}\rangle \langle \lambda_{i}|\psi \rangle +\int \langle \phi |\lambda_{\rho }\rangle \langle \lambda_{\rho }|\psi \rangle \rho \left(\lambda \right)\;d\lambda . \end{array}$$

Therefore, $|\xi_{\lambda }\rangle \langle \xi_{\lambda }|\;d\mu \left(\lambda \right)=|\lambda_{\rho }\rangle \langle \lambda_{\rho }|\rho \left(\lambda \right)\;d\lambda$ (ρ⩾0, integrable), that is, we may say that $\left|\lambda \rangle =\right|\lambda_{\rho }\rangle \sqrt{\rho (\lambda )}$ are Dirac’s kets and $\sqrt{\rho (\lambda )}\;\langle \lambda |=\langle \lambda_{\rho }|$ are Dirac’s bras.

### 5.3. Spectral Projections

According to von Neumann, there is a decomposition into a direct integral

$$H≃\int_{R}^{⊕}H\left(\lambda \right)\;d\mu \left(\lambda \right),$$

that “decomposes” *A*:

$$\begin{array}{cc} f & ∼\left\{f\left(\lambda \right)\right\},\;f\left(\lambda \right)\in H\left(\lambda \right),\;\mathrm{with}{∥f∥}_{}^{2}=\int_{R}{\left|f\left(\lambda \right)\right|}^{2}\;\;d\mu \left(\lambda \right),\\ Af & ∼\{\lambda f(\lambda )\}. \end{array}$$

The problem is that H(λ) is not a subspace of H if λ is a point of null μ-measure. Thus, there are no true eigenvectors corresponding to points of the continuous spectrum.

If Φ is nuclear, the map $\tau_{\lambda }:\phi \mapsto \phi \left(\lambda \right),\;\phi \in \Phi ,\;$ϕ(λ)∈H(λ), is continuous and nuclear for μ–almost all λ. Thus one may write

$$\tau_{\lambda }\phi =\phi \left(\lambda \right)=\langle \phi |\xi_{\lambda }\rangle h\left(\lambda \right),\mathrm{where}\xi_{\lambda }\in \Phi^{\times },\;h\left(\lambda \right)\in H\left(\lambda \right).$$

Therefore, the dual map $\tau_{\lambda }^{\prime }:H\left(\lambda \right)\to \Phi^{\times }$ is also continuous, so that

. one may identify every vector ξ∈H(λ) with a functional $\tilde{\xi }=\tau_{\lambda }^{\prime }\xi \in \Phi^{\times }$
. $\chi_{\lambda }=\tau_{\lambda }^{\prime }\tau_{\lambda }$ is a nuclear operator from Φ into $\Phi^{\times }$; projection on the eigenspace $\Phi_{\lambda }^{\times }$ corresponding to the eigenvalue λ.

However, there is a difficulty here: the spectrum of *A* in $\Phi^{\times }$ may contain points that do not belong to the Hilbertian spectrum, a situation to be avoided.

To give an example: Take the momentum operator $p=\frac{1}{i}\frac{\;d}{\;dx}$; then, its spectrum σ(p) is

$$\begin{array}{cc} \;.\;\;\sigma (p) & =R\;\;\mathrm{in}\;S\subset L^{2}\subset S^{\times },\\ .\;\;\sigma (p) & =C\;\;\mathrm{in}\;D\subset L^{2}\subset D^{\times }. \end{array}$$

Fortunately, there are criteria for ensuring that one gets a tight rigging [25], that is, a rigging in which the RHS spectrum coincides with the Hilbertian spectrum.

The standard example of a RHS analysis is that of a particle, either free or in sufficiently regular potential *V*: The observables are: position Q, momentum P, energy $H=-P^{2}/2m+V\left(Q\right)$, and the natural RHS is the Schwartz triplet

$$S\left(R^{3}\right)\subset L^{2}\left(R^{3}\right)\subset S^{\times }\left(R^{3}\right).$$

## 6. Conclusions

The conclusion of this analysis is that von Neumann’s approach to QM is necessary, but not sufficient. The RHS approach completes the latter and allows a rigorous version of the practical formalism of Dirac. It leads to a good formulation of scattering theory: plane waves, Gamow states, resonances, Lippman–Schwinger equation, etc. In addition, it extends naturally to quantum field theory (Borchers, Wightman,...).

An additional bonus is that the RHS approach provides a good realization of symmetries. Let *U* be a unitary representation of an invariance group *G* in H. Then, we obtain two additional representations of *G*:

. $U_{\Phi }$ acting in Φ (active point of view)
. $U_{\Phi }^{\times }$ acting in $\Phi^{\times }$ (passive point of view)

and these two representations are contragredient of each other, as consequence of the unitarity of *U* in H):

$$\langle U_{\Phi }\left(g\right)\phi |U_{\Phi }^{\times }\left(g\right)F\rangle =\langle \phi |F\rangle ,\;\forall \;g\in G,\phi \in \Phi ,F\in \Phi^{\times }.$$

Before quitting, let me give four more references, which are particularly relevant to the present paper. First, a general discussion of the RHS approach may be found in the so-called Compendium of Quantum Physics [26]. For going further in the philosophical implications of QM and its various interpretations, an invaluable source is the classical book by Jammer [27], who also makes contact with the quantum logic, involving nondistributive lattices.

Next, the textbook by Hannabuss [28] gives a clear and pedagogical treatment of QM, suitable for 2nd or 3rd year mathematics students. Notably, it contains a remarkable collection of original citations.

Finally, one should quote the book by Lacki [29]. He analyzes von Neumann’s book as concretization of Hilbert’s program on the axiomatization of physics, already undertaken by Jordan and Nordheim. In particular, he analyzes in detail Neumann’s “proof” of the impossibility of hidden variables in QM.

However, this is not the end of the story. All we have said concerns the standard approach to QM, valid for a single system, in its successive formulations: von Neumann’s, Dirac’s, and the RHS version. In the background, this still relies in some form of the Copenhagen interpretation. An extension to a collection of identical systems, which is the realm of statistical physics, is readily available.

First, QM has evolved into what is called the standard model of elementary particles. This is certainly not the final word, although it has passed successfully all comparisons with experiment. Then, new extensions of QM have appeared in the recent years, trying to combine it in a rigorous manner with gravity and also information theory. Of course, this is not the place to go into detail. Instead, we might redirect the reader to the fascinating little book by Al-Khalili [30] for a thorough, but nontechnical panorama of the recent evolution of physics. Of course, all these recent developments go far beyond our considerations in the present paper, but nevertheless, these have a nontrivial place in the theory: mathematical rigor is not a luxury, notwithstanding Pauli’s opinion quoted above.
